# Operating length and velocity of human vastus lateralis muscle during walking and running

**DOI:** 10.1038/s41598-018-23376-5

**Published:** 2018-03-22

**Authors:** S. Bohm, R. Marzilger, F. Mersmann, A. Santuz, A. Arampatzis

**Affiliations:** 1 Department of Training and Movement Sciences, Humboldt-Universität zu Berlin, Berlin, Germany; 2 Berlin School of Movement Science, Berlin, Germany

## Abstract

According to the force-length-velocity relationships, the muscle force potential during locomotion is determined by the operating fibre length and velocity. We measured fascicle and muscle-tendon unit length and velocity as well as the activity of the human vastus lateralis muscle (VL) during walking and running. Furthermore, we determined the VL force-length relationship experimentally and calculated the force-length and force-velocity potentials (i.e. fraction of maximum force according to the force-length-velocity curves) for both gaits. During the active state of the stance phase, fascicles showed significantly (p < 0.05) smaller length changes (walking: 9.2 ± 4.7% of optimal length (L_0_); running: 9.0 ± 8.4%L_0_) and lower velocities (0.46 ± 0.36 L_0_/s; 0.03 ± 0.83 L_0_/s) compared to the muscle-tendon unit (walking: 19.7 ± 5.3%L_0_, −0.94 ± 0.32 L_0_/s; running: 34.5 ± 5.8%L_0_, −2.59 ± 0.41 L_0_/s). The VL fascicles operated close to optimum length (L_0_ = 9.4 ± 0.11 cm) in both walking (8.6 ± 0.14 cm) and running (10.1 ± 0.19 cm), resulting in high force-length (walking: 0.92 ± 0.08; running: 0.91 ± 0.14) and force-velocity (0.91 ± 0.08; 0.97 ± 0.13) potentials. For the first time we demonstrated that, in contrast to the current general conception, the VL fascicles operate almost isometrically and close to L_0_ during the active state of the stance phase of walking and running. The findings further verify an important contribution of the series-elastic element to VL fascicle dynamics.

## Introduction

A muscle’s potential to generate force depends on intrinsic muscle properties as the force-length and force-velocity relationships^1,2^. Consequently, the operating length and velocity of the muscle fibres during locomotion determine the force potential of the muscle, i.e. the fraction of maximum force according to the force-length and force-velocity curves. Roberts *et al*.^3^ measured muscle fibre length and tendon force in the lateral gastrocnemius muscle of running turkeys *in vivo* and found that during the stance phase of running, where the muscle was active and generated force, the muscle fibres operated with almost constant length (i.e. maximum length change of 6.6% of the optimal length (*L*_*0*_)) and close to *L*_*0*_. The nearly isometric contraction of the muscle fibres was a result of a concomitant lengthening of the tendon that provided more than 60% of the work done by the muscle-tendon unit (MTU). Hence, the decoupling of muscle and tendon lengthening that enabled favourable muscle contraction conditions was concluded to be beneficial for economic force generation, since less muscle volume needs to be activated for a certain force output^3^.

In the past two decades, numerous studies investigated the fascicle behaviour of the human distal leg muscles, as for example soleus and gastrocnemius medialis, using the ultrasound methodology during walking and running *in vivo*^4–7^. A decoupling of fascicle and tendon length changes during both gaits, which resulted in comparably smaller length changes at the fascicle level compared to the MTU, was consistently reported. However, for the human proximal leg muscles, as for example the vastus lateralis (VL), information regarding the fascicle behaviour during walking and running is scarce. It has been suggested that proximal muscles feature less compliant tendons and, therefore, MTU’s length changes would be accompanied by major changes in fascicle length^8–10^. In this regard, modelling studies predicted a stretch-shortening cycle of the VL muscle during the stance phase with notable fascicle length changes of up to ≈25% of optimal length during running^11^ and ≈20% during walking^11,12^, covering a wide portion around the plateau of the force-length curve. However, experimental investigations demonstrated that the strain of the VL tendon-aponeurosis during a maximal isometric voluntary contraction *in vivo* is comparable to the strain of the gastrocnemius medialis’ tendon aponeurosis^13,14^ and evidently higher than the strain values used in the mentioned modelling studies (≈8.0% vs. 3.3% maximal strain). Consequently, the predicted VL fascicle length changes during walking^11,12,15^ and running^11^ might be overestimated^12^. Moreover, in a recent study we reported a facilitation of the force-velocity and power-velocity potentials of the VL muscle due to tendon compliance during vertical jumping and provided evidence for an important contribution of the series elastic tendon to the VL muscle force and power production^16^.

The muscle activation pattern (onset, duration and amplitude) can affect the interaction within the MTU and, thus, the operating length and velocity of the fascicle^17–20^. By adjusting the stimulation onset timing during a work loop paradigm of an isolated bullfrog’s plantaris MTU subjected to cyclic strain, Sawicki and colleagues (2015)^18^ were able to influence the degree of decoupling of muscle fascicle and MTU trajectories. Only a specific stimulation onset timing condition led to zero net mechanical work of the MTU, which compares to terrestrial locomotion, featuring relatively small length changes of the fascicles and consequently lower fascicle shortening velocity compared to the MTU^18^. Moreover, when the cyclic contractions were matched with the natural frequency of a biological MTU in a more unconstrained work loop, this resulted in a naturally emerging spring-like behaviour with maximal muscle forces and maximal fractions of mechanical work performed by the series elastic elements^20^. Although *in vitro* studies may not directly reflect the complex interactions during human locomotion *in vivo*, apparently the muscle activation pattern plays a key role for the interaction of the MTU components^21^ and an adjusted activation pattern may facilitate fascicle operating conditions towards high force potentials.

The purpose of the current study was to experimentally investigate the fascicle’s operating length and velocity as well as the activation of VL during human steady-state level walking and running. We measured the force-fascicle length relationship of the VL muscle by maximal voluntary isometric contractions at different knee joint angles and assessed the maximal shortening velocity of the VL using muscle-specific parameters that were provided by literature reports^22^. We hypothesised that the VL muscle would demonstrate small fascicle length changes close to *L*_*0*_ during level walking and running. We expected that the muscle intrinsic mechanics (i.e. force-length-velocity relationships) are governed by adjusted muscle activation in a way that facilitates the VL force generation potential during the different locomotion modes, i.e. walking and running. Furthermore, we evaluated the reliability of the ultrasound-based VL’s fascicle length determination during walking and running by a systematic test-retest analysis, since such methodologically essential information has not been provided yet.

## Methods

### Experimental design

Thirty healthy adults (5 female) participated in the present study after giving written informed consent to the experimental procedure, which was approved by the local ethics committee (Ethikkomission, Ethikausschuss 2 am Campus Virchow-Klinikum, Charitéplatz 1, 10 117 Berlin; reference number EA2/076/15) and in accordance with relevant guidelines and regulations. The participants were regularly physically active and did not report any history of neuromuscular or skeletal impairments in the past six months. The participants were equally distributed to two groups (i.e. n = 15). In the first group (age: 27.3 ± 4.1 yrs., height: 179.2 ± 6.5 cm, mass: 75.0 ± 8.2 kg), the individual force-fascicle length relationship of the VL muscle was experimentally assessed by means of maximal isometric voluntary knee extensions contractions (MVC) of the right leg at different knee joint angles on a dynamometer in combination with ultrasound imaging of the VL fascicles. The force applied to the patellar tendon was calculated from the knee joint moment and the tendon lever arm, which was determined by magnetic resonance imaging (MRI). On a second day, the VL fascicle length and the joint kinematics of the same leg were measured synchronously during walking (1.5 m/s) and running (3.0 m/s) on a treadmill using ultrasonography and motion analysis, respectively. Walking and running order was randomised between participants and a two-minute warm-up and familiarisation phase for each speed preceded the 10-minute trials. The measurements were repeated on a following day for a reliability analysis. The participants of the second group (age: 29.3 ± 6.7 yrs., height: 176.9 ± 8.0 cm, mass: 71.0 ± 12.0 kg) performed the same walking and running protocol on the treadmill while electromyographic (EMG) activity of the right VL muscle was captured. The VL activity data was then combined with the kinematics and fascicle length data of the first group.

### Assessment of muscle intrinsic properties

The participants were seated on a dynamometer (Biodex Medical, Syst. 3, Inc., Shirley, NY), fixed with a pelvic strap around the waist while the arms were held crossed above the chest. The hip joint angle was set to 85° (0° = supine) in order to reduce the contribution of the bi-articular m. rectus femoris to the knee extension moment^23^. Following a standardised warm-up, eight MVCs of the right leg including a plateau of around 2 s were performed in a range of 20° to 90° knee joint angle (0° = knee extended) in 10° intervals in a randomised order. As the angles based on the dynamometer data during rest are not representative for the knee angles during contractions due to soft tissue deformation and dynamometer compliance^24^, leg kinematics were recorded on the basis of six reflective markers (anterior iliac spine, greater trochanter, lateral and medial femoral epicondyle and malleoli) using a Vicon motion capture system (Version 1.7.1., Vicon Motion Systems, Oxford, UK) integrating eight cameras (6x F20, 2x T20) at 250 Hz. Marker trajectories were smoothed using a second-order low-pass Butterworth filter with a cut-off frequency of 6 Hz^16^.

The resultant moments at the knee joint were calculated by means of inverse dynamics according to the methodology reported by Arampatzis *et al*.^24^ to account (a) for the effect of the misalignment between knee joint axis and dynamometer axis and (b) the effect of the gravitational forces. Accordingly, joint angle-specific moments due to gravity were determined during a passive knee joint rotation (5°/s) driven by the dynamometer, while the participants were completely relaxed. Furthermore, the contribution of the antagonistic moment produced by the hamstring muscles was considered by establishing a relationship between EMG amplitude and exerted moment of the hamstrings while working as agonist^25^. The EMG activity of the muscle biceps femoris and the corresponding moment produced by the hamstrings was measured in a relaxed condition and during two additional submaximal isometric knee flexion contractions of different intensity, according to the methodology reported by Mademli *et al*.^26^. The EMG activity was measured synchronously with the kinematic data using a wireless EMG system (Myon m320RX, Myon AG, Baar, Switzerland) at an acquisition frequency of 1000 Hz.

The force applied to the patellar tendon during the MVCs was calculated as quotient of the knee joint moment and the tendon lever arm. For the fully extended knee, the lever arm was measured in a three-dimensional coordinate system as the perpendicular distance of the tendon’s line of action to the rotation axis of the knee based on MRI. The line of action of the patellar tendon was defined as the line of best linear fit through the geometrical centres of the tendon cross-sectional areas, which were reconstructed from the segmentation of transverse images (G-Scan, 0.25 T, 3D HYCE (GR) sequence, Esaote, Genova, Italy) between the caudal pole of the patellar bone and the initial insertion at the tibial tuberosity. The corresponding axis of rotation of the knee joint was determined by segmenting the lateral and medial femoral epicondyles in the sagittal magnetic resonance scans and connecting the centres of the respective best fitting circles according to Churchill *et al*.^27^. The tendon moment arm as a function of the knee joint angle was calculated by processing moment arm changes in relation to joint angle on the basis of the data provided by Herzog and Read^28^.

**Figure 1:**
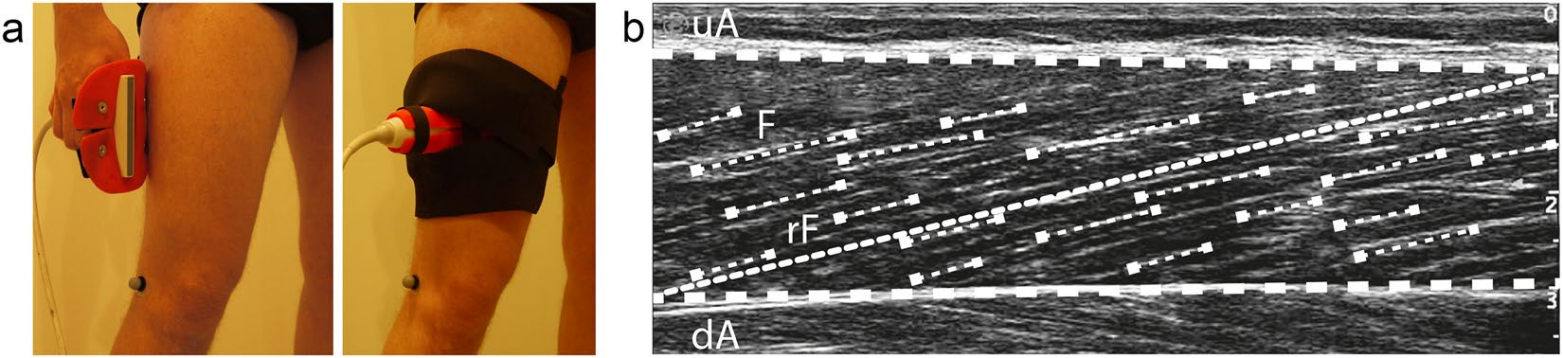
Ultrasound-based determination of vastus lateralis muscle fascicle length. (**a**) The ultrasound transducer was carefully attached on the muscle belly by means of a customised neoprene elastic cast. (**b**) A semi-automatic frame-by-frame tracking procedure was used to identify visible features of multiple fascicles (F) located between the manually traced upper (uA) and deeper (dA) aponeurosis. A representative reference fascicle (rF) was then calculated on the basis of the identified fascicle portions to assess fascicle length as the Euclidian distance between the insertion points with the two aponeurosis.

**Figure 2:**
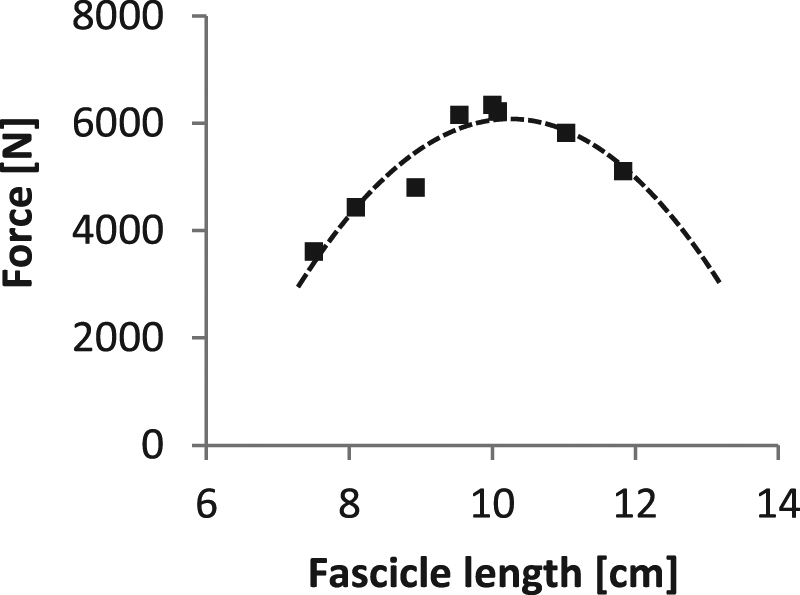
Exemplary force-fascicle length relationship of the vastus lateralis muscle. The force-fascicle length relationship was experimentally determined by means of maximal voluntary isometric knee extensions (squares) and a respective second-order polynomial fit (dashed line).

During the MVCs the VL fascicles were captured by B-mode ultrasonography (My Lab60, Esaote, Genova, Italy). A 10 cm linear array probe operating at 43 Hz (LA923, 10 MHz, depth 7.4 cm, focal point 1.8, no image filter) was attached to the skin above the VL muscle belly (≈50% of femur length), adjusted with respect to parallel superficial and deeper aponeurosis and clarity of aligned hyperechoic perimysial intramuscular connective tissue that is indicative for the muscle fascicle structures, and fixed by elastic straps. The ultrasound device and motion capture system were synchronised by a manually-released 5 V trigger signal. The fascicle length was determined from the ultrasound videos by a self-developed semi-automatic tracking algorithm^29^ written in Matlab (version R2012a, The Mathworks, Natick, USA). Briefly, the procedure included an approximation of the deeper and superficial aponeurosis by a best linear fit through three manually placed and frame-by-frame adjusted marks on the respective inner connective tissue layer (Fig. 1). Then, a semi-automated algorithm based on the *bwtraceboundary* function of the Matlab Image Processing toolbox automatically identified the shape and orientation of image brightness features between both aponeurosis in each frame, which are indicative for the hyperechoic perimysial connective tissue parts (snippets) aligned with the muscle fascicles (Fig. 1). Detected snippets were considered as valid the following requirements were fulfilled: minimal length of 23 pixels (i.e. 0.4 cm, from the bottom left to the top right point of a snippet); area to length ratio of 8.5 (identifies white areas with a long and narrow shape that were then converted to lines); angle between snippet and upper aponeurosis between 6° and 35°; 80% of the pixels on a line between the start and end point of a snippet had to be white. Every frame was visually controlled afterwards for adequate feature placement and manually corrected if necessary (e.g. non-perimysial connective tissue portions were removed). A linear reference fascicle was calculated as an average of the single identified features and was used for fascicle length determination (Fig. 1). Furthermore, the fascicle length was averaged over ten frames from the plateau of each MVC. On the basis of the maximum force applied to the patellar tendon and the corresponding VL fascicle length, an individual force-fascicle length relationship was calculated for each participant based on a second-order polynomial fit (Fig. 2), to determine the maximum muscle force applied to the tendon (*F*_*max*_) and the *L*_*o*_ for force generation. VL muscle specific constants of *a*_*rel*_ = *0*.*34* and *b*_*rel*_ = *4*.*03* *s*^*−1*^ ^22^ were used to assess the maximal fascicle shortening velocity *V*_*max*_ = 11.85 *L*_*0*_ *s*^*−1*^. The force-velocity relationship of the VL fascicles was then described following the classical Hill equation^2^.

### Assessment of joint kinematics and muscle fascicle length during walking and running

During 10-minute walking and running trials on the treadmill (Daum electronic, ergo_run premium8, Fürth, Germany), kinematic data of the right leg were recorded by the Vicon motion capture system (5x Vicon MX T20, 5x Vicon MX-T20-S, 250 Hz), using anatomically-referenced markers placed on the greater trochanter, lateral femoral epicondyle, lateral malleolus, head of second metatarsalis and tuberositas calcanei. The touchdown of the foot during walking and running was determined from the kinematic data as instant of minimal vertical position of the heel marker^30,31^, the toe-off during walking as reversal of horizontal velocity of the metatarsalis marker^30,32^ and during running as the minimum in knee joint angle, i.e. most extended knee position^31^.

During the respective 10 minutes of walking and running, an ultrasound recording of 10 s was captured synchronously with the kinematic data every two minutes. While the data of one trial was used to compare the VL fascicle behaviour between gaits, all five trials were used for the reliability analysis (see below). The ultrasound images were recorded at a capture frequency of 43 Hz using a 10 cm linear array transducer that was fixed in a custom-made flexible, antiskid neoprene/plastic cast (Fig. 1) and fascicle length was measured as described above. Fascicle length data was filtered using a second-order low-pass Butterworth filter with a cut-off frequency of 6 Hz and averaged over 6 to 11 steps (8.1 ± 0.9) for each participant and gait (i.e., walking and running).

**Figure 3:**
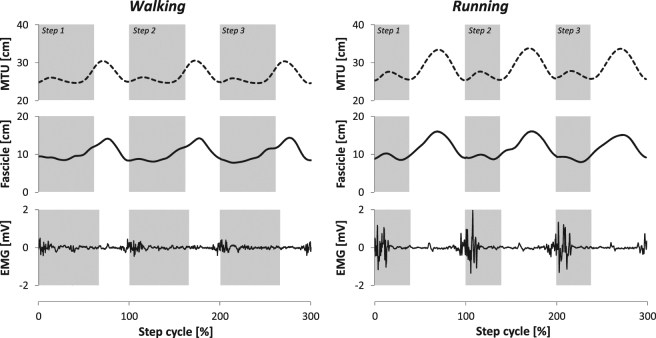
Vastus lateralis muscle-tendon unit (MTU) length, muscle fascicle length and electromyographic (EMG) activity of a representative participant during three consecutive walking (left) and running (right) steps. Respective stance phases are shaded in grey.

The associated length change of the VL MTU during locomotion was calculated as the product of the change in knee joint angle and the individual angle-specific patellar tendon lever arm^33^. The initial MTU length at touchdown of the foot was determined based on the regression equation provided by Hawkins and Hull^34^. MTU and fascicle velocities during locomotion were calculated as the first derivative of the length change over time. Figure 3 illustrates the length changes of the VL fascicles and MTU during walking and running from a representative participant over three consecutive step cycles.

The experimental determination of the VL fascicle length during locomotion was tested for reliability by comparing five single assessments on two separate days (3 to 4 days in between). To achieve a precise repositioning of the ultrasound probe on the second measurement day the four corner points of the ultrasound probe were marked on the skin using a non-permanent marker. The marked positions were measured with a flexible measurement tape in respect to the medial and lateral femur condyles, representing fixed anatomical landmarks. On the second day, the position was reconstructed accordingly and the participants performed the same gait protocol while again the fascicle length was captured five times.

### Assessment of EMG muscle activity during locomotion

Surface EMG of the right VL muscle was measured during the walking and running trials after eight minutes on the treadmill for 60 s by means of the wireless EMG system and two bipolar surface electrodes (2 cm inter-electrode distance) that were placed on the muscle belly. A second-order high-pass Butterworth filter with a 20 Hz cut-off frequency, a full-wave rectification and then a low-pass filter with a 20 Hz cut-off frequency were applied to the raw EMG data. The EMG activity was averaged over 10 walking and running steps, respectively, and normalised for each participant to the maximum value achieved during running. To determine the onset of the VL muscle activity during walking and running we used a threshold that was defined as the baseline activity plus three times its standard deviation^35,36^. A representative raw EMG data set is presented in Fig. 3.

### Statistics

The stance and swing phases of each step cycle of the EMG group were separately time-normalised to those of the fascicle group to be able to relate the EMG with the fascicle and MTU parameters from the two different groups. A two-way analysis of variance (ANOVA) for repeated measures was conducted to test the parameters (absolute and normalised to *L*_*o*_ fascicle and MTU length, respective operating ranges and EMG activation state duration) for activation state (active vs. inactive state) and gait condition (walking vs. running) effects. A two-way repeated measures ANOVA was also used to test parameters (normalised fascicle and MTU velocity) for component (fascicle vs. MTU) and gait condition (walking vs. running) effects during the active state of the stance phase. The normality of the standardised residuals of all investigated parameters was tested by the Shapiro-Wilk test using the respective ANOVA model. Interaction effects were tested post-hoc by a paired t-test for the differences of the respective variable values. A paired t-test (two-tailed) was used to test for differences of averaged EMG activity, normalised fascicle length and velocity as well as force-velocity potentials in the active state between walking and running. In case of non-normality of the residuals (absolute and relative range of fascicle length changes, force-length potential) the Wilcoxon signed-rank test was applied accordingly. Group anthropometrics were compared by means of a t-test for independent samples.

The coefficient of multiple correlations (CMC)^37^ was used to test the reliability of the fascicle length determination for the entire step cycle of the five trials on the two days. Root mean square differences (RMSD) were calculated for day 1 and 2 and both days to quantify the variability between trials. An ANOVA for repeated measures was performed to examine possible differences in the gait cycle between the two test days for walking and running. The level of significance was set at α = 0.05. The α-level was adjusted to 0.025 for the post-hoc analysis as well as separate non-parametric testing of the two factors.

### Availability of materials and data

The datasets generated and analysed during the current study are available from the corresponding author on reasonable request.

## Results

Body height, mass and age was not statistically significant between groups. The experimentally assessed *L*_*o*_ was in average 9.43 ± 1.12 cm and corresponding *F*_*max*_ was 4918 ± 923 N. The assessed *V*_*max*_ based on the reported values of *a*_*rel*_* = *0.34 and *b*_*rel*_ = 4.03 s^−122^ was 111.7 ± 13.3 cm s^−1^. The reliability analysis for the fascicle length determination revealed high within-day (walking 0.952 ± 0.016, running 0.957 ± 0.016), between-day (walking 0.894 ± 0.091, running 0.916 ± 0.13) and overall CMCs (walking: 0.905 ± 0.048, running 0.921 ± 0.063). The RMSD for walking were 6.8 ± 1.3 mm for day 1, 7.3 ± 1.1 mm for day 2 and 7.4 ± 0.7 mm for both days and for running 7.2 ± 1.3 mm for day 1, 7.5 ± 1.3 mm for day 2 and 7.6 ± 0.8 mm for both days. No measurement day effect was found (walking p = 0.325, running p = 0.281).

**Figure 4:**
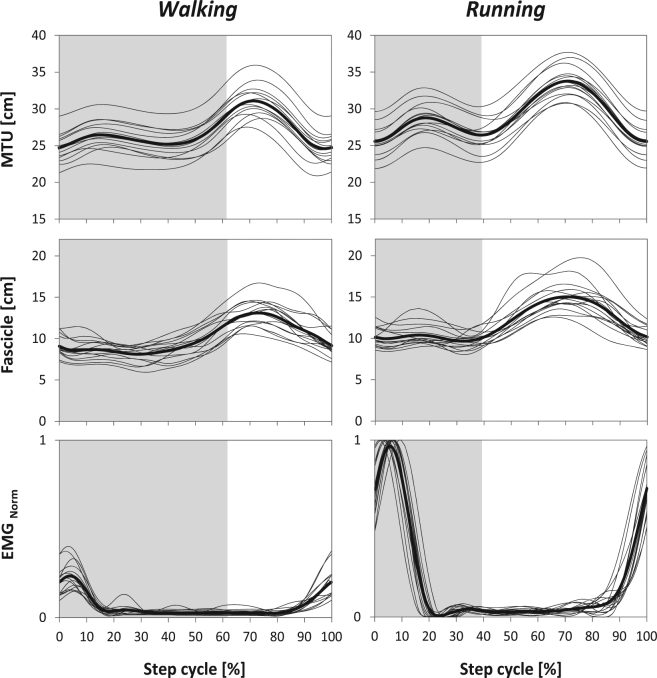
Vastus lateralis muscle-tendon unit (MTU) length, muscle fascicle length and electromyographic (EMG) muscle activity. Individual (n = 15) data are shown in thin grey lines and means in thick black lines separately for the walking (left column) and running (right column) step cycle. EMG activity was normalised to the maximum achieved during running. Grey shadings refer to the stance phase.

**Figure 5:**
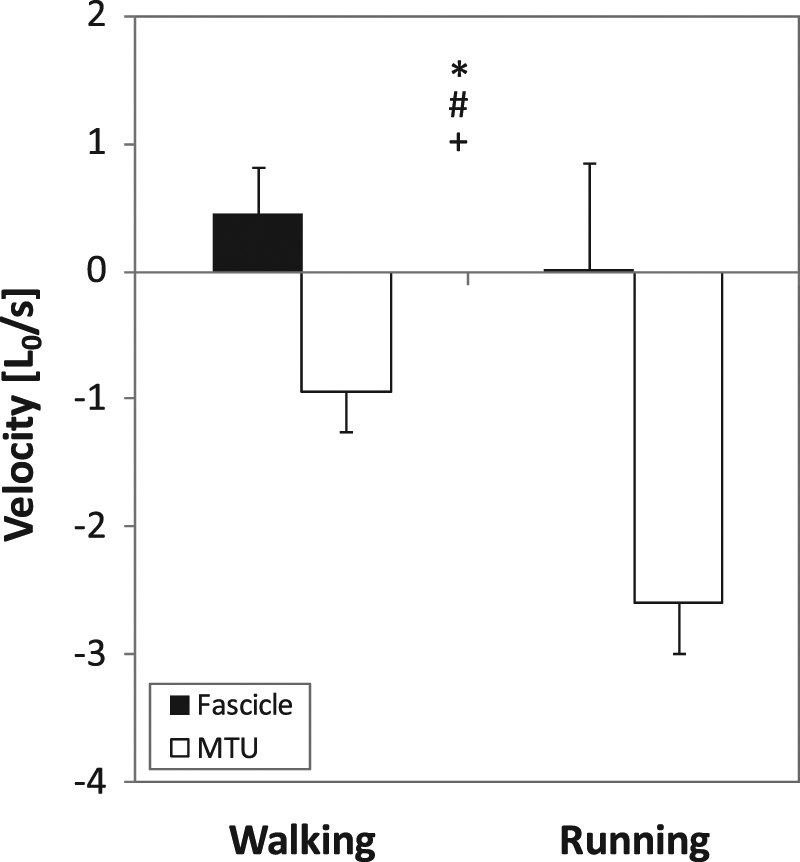
Vastus lateralis muscle-tendon unit (MTU) and muscle fascicle velocity normalised to optimal fascicle length (*L*_*0*_) during the active state of the stance phase while walking and running (mean and standard deviation; n = 15). *Statistically significant effect of component (fascicle vs. MTU, p < 0.05). ^#^Statistically significant effect of gait condition (walking vs. running, p < 0.05). ^+^Statistically significant component *x* gait condition interaction (p < 0.05).

The averaged stance and swing times were 663 ± 51 ms and 382 ± 28 ms for walking and 301 ± 30 ms and 434 ± 32 ms for running. The VL muscle was active during the first 19.0 ± 6.1% of the gait cycle in walking and during the first 18.6 ± 2.0% in running (Table 1, Fig. 4). The peak EMG activity occurred at 4.8 ± 1.6% of the gait cycle for walking and 6.4 ± 2.2% for running. The muscle was then inactive until 85.1 ± 2.9% of the gait cycle for walking and 85.2 ± 4.1% for running and became active again prior touchdown (Fig. 4). The EMG activity was significantly higher in running compared to walking (p < 0.001, Table 1). During the stance phase, where the VL muscle was active, the length changes of the fascicles were significantly smaller compared to the MTU length changes (walking: 9.2 ± 4.7%*L*_*o*_ vs. 19.7 ± 5.3%*L*_*o*_, running: 9.0 ± 8.4%*L*_*o*_ vs. 34.5 ± 5.8%*L*_*o*_, p < 0.001). Accordingly, the average fascicle velocity during the active state was significantly lower compared to the MTU velocity (Fig. 5). Substantial length changes at the fascicle level occurred only in the phase where the muscle was inactive (walking 57.1 ± 9.7%*L*_*o*_ and running 61.4 ± 17.1%*L*_*o*_, p = 0.47, Table 1). The ratio (running to walking) of the averaged VL fascicle velocity and VL MTU velocity (absolute values) during the active state was significantly lower on the fascicle level (0.75 ± 0.45 vs. 3.06 ± 1.11, p < 0.001).

**Table 1:** Vastus lateralis average muscle-tendon unit (MTU) length and MTU length changes, average fascicle length and fascicle length changes, average electromyographic (EMG) activity and duration of the active and inactive muscle state (mean and standard deviation, n = 15). Note that the muscle active state was examined during the stance phase and the inactive state with respect to the entire step cycle.

Parameter	Active		Inactive	
	Walking	Running	Walking	Running
MTU length (cm)^a,b,c^	25.81 ± 1.95	27.17 ± 2.02	27.46 ± 2.02	29.82 ± 1.94
MTU length change (cm)^a,b^	1.81 ± 0.37	3.21 ± 0.40	6.01 ± 0.45	7.40 ± 1.15
Fascicle length (cm)^a,b,c^	8.63 ± 1.14	10.14 ± 1.19	10.24 ± 1.28	12.29 ± 1.35
Fascicle length change (cm)	0.87 ± 0.50	0.85 ± 0.82	5.34 ± 0.85^+^	5.73 ± 1.57^+^
EMG activity_Norm_	0.153 ± 0.058	0.675 ± 0.027*	—	—
State duration (ms)^a,b,c^	201 ± 67	136 ± 18	695 ± 78	486 ± 32

^a^Statistically significant effect of gait condition (p < 0.05).

^b^Statistically significant effect of activation state (p < 0.05).

^c^Statistically significant gait condition x activation state interaction (p < 0.05).

^+^Statistically significant difference between active and inactive state (p < 0.05).

^*^Statistically significant difference between walking and running (p < 0.05).

**Figure 6:**
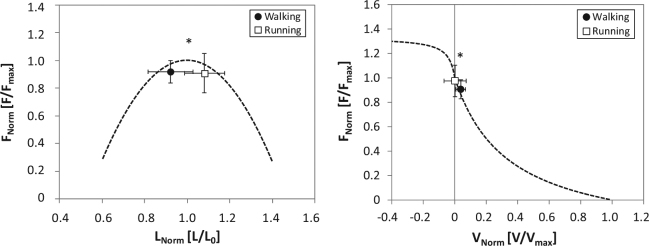
Operating length and velocity of vastus lateralis muscle fascicles during the active state of the stance phase during walking and running onto the normalised force-length and force-velocity curve (mean and standard deviation; n = 15). Force is normalised to the maximum force obtained during the maximal isometric knee extension contractions, fascicle length to the experimentally-determined optimal fascicle length and fascicle velocity to the estimated maximum shortening velocity. *Statistically significant differences between walking and running (p < 0.05).

The average length of the VL MTU and fascicles were significantly longer in running compared to walking in both active and inactive muscle state (Table 1). However, in both gaits the fascicles operated very close to their optimal length (relative values to *L*_*0*_ for walking 0.92 ± 0.10 and running 1.08 ± 0.09) and with very low contraction velocities (relative values to *V*_*max*_ for walking 0.039 ± 0.031 and running 0.002 ± 0.070, Fig. 6) during the stance phase where the muscle was active. Accordingly, the force-length and force-velocity potentials of the VL muscle were quite high, i.e. force-length potential 0.92 ± 0.08 and 0.91 ± 0.14 (p = 0.691) and force-velocity potential 0.91 ± 0.08 and 0.97 ± 0.13 (p = 0.027) for walking and running, respectively.

## Discussion

The present study experimentally investigated the operating length and velocity of the human VL fascicles during level walking and running. The findings provide evidence that the fascicles of the VL operate close to their optimum working length with very small length changes (i.e. almost isometrically) in the stance phase, where the muscle is active and generates force. The central nervous system seems to control the operating length and velocity of the VL fascicles by adjusting timing and magnitude of muscle activation to maintain high force potentials considering the force-length and force-velocity relationships. Based on these findings our hypothesis was confirmed.

During walking and running the VL was activated in the initial stance phase, which is associated with the function of the muscle to decelerate and support the body mass^38,39^. During the active state, the MTU showed a notable elongation (walking 20% and running 35% of *L*_*o*_), whereas the fascicles operated with significantly smaller length changes (9% of *L*_*o*_ for walking and running) and close to their optimal length. The high differences between MTU and fascicle length changes demonstrate that the main length changes of the MTU were primarily associated with changes of the series-elastic elements of the VL (patellar and quadriceps tendon complex). These findings provide evidence that tendon elasticity significantly contributed to the VL MTU function, resulting in a considerable decrease of the fascicle velocity. This decoupling of fascicle and MTU length trajectories allowed the fascicles to take advantage of the high force-length-velocity potentials during the active phase of the stance phase where the VL muscle generates force. Substantial changes in fascicle length were only found during the inactive state. Further, the ratio (running to walking) of the averaged VL fascicle velocity and VL MTU velocity was lower on the fascicle level, indicating a pronounced contribution of tendon elasticity to the fascicle behaviour during running.

In contrast to the present findings, modelling studies on VL fascicle behaviour during walking and running predicted a stretch-shortening cycle of the VL fascicles, with significant fascicle length changes of ≈25% of optimal length during running^11^ and ≈20% during walking^11,12^. These studies implemented a highly stiff VL tendon in their musculoskeletal model, i.e. 3.3% strain at maximum muscle force. The functional consequence of an overestimated VL tendon stiffness during walking and running is an increase of the fascicle length change to MTU length change ratio at a given muscle activation that results in higher fascicle velocities. *In vivo* studies reported a patellar tendon strain of 6 to 8%^40–43^ and VL tendon of 8 to 9%^44,45^ during maximal isometric contractions. These strain values are by far greater as the strain characteristics of the tendons implemented in the musculoskeletal models^11,12^, indicating unrealistic predictions of length changes at the fascicle level compared to the MTU. The current experimental results of the VL function during walking and running clarify that tendon elasticity substantially influences the fascicle length changes and, thus, the contractile efficiency of human proximal leg muscles.

The favourable operating conditions (i.e. minimal length changes of VL fascicle and close to optimal length) seem to be the result of adjusted muscle activation. In both walking and running, the VL muscle was activated prior touchdown and EMG activity increased in the first part of the stance phase where the MTU is lengthened. The coordinated time course of the EMG activity and MTU elongation provide evidence that time-adjusted muscle activation contributes to the minimisation of fascicle length changes during the stance phase of walking and running. The consequences are high potentials to generate force due to the force-length and force-velocity relationships in both gaits. Our results indicate that by means of an appropriate activation tendon elasticity is used for the simplification/linearisation (i.e. by small operating length and velocity ranges) of a complex system (VL MTU) with non-linear properties as the force-length and force-velocity relationship during walking and running. Relative to the step cycle, the VL EMG activity profile was quite similar between walking and running (no significant gait effect) but with significantly higher values during running. It can be argued that the higher activation of the VL muscle in the first part of the stance phase during running, at similar force-length and force-velocity potentials, resulted in higher muscle force and, thus, greater elongation of the VL tendon compared to walking. The greater elongation of the VL tendon due to greater muscle activation, can explain the observed lower running to walking fascicle velocity ratio compared to the MTU velocity ratio and, thus, the pronounced contribution of tendon elasticity on fascicle behaviour in running.

The favourable fascicle operating conditions observed in the present study may have functional importance for human locomotion because a less active muscle volume is required for a certain mechanical demand, allowing for a reduction in metabolic cost during locomotion^3,46^. This might be of particular importance since the quadriceps muscle group is quite voluminous and features longer fascicles compared to the other lower limb muscles^47–49^. Modelling approaches^38,39^ as well as experimental data^50^ indicate that the main function of the VL during walking and running is to decelerate and support the body mass in the first part of the stance phase. In the following propulsion phase, the plantar flexor muscles provide the main support and acceleration of the body mass, while the contribution of the VL is marginal^38,39,50^. The functional consequence of a minimal elongation of the VL fascicles (i.e. more or less isometric contraction) in the first part of the stance phase is a minimisation of energy dissipation by the contractile element of the muscle during the deceleration of the centre of mass. The series-elastic element of the VL muscle takes over the elongation of the MTU and the energy needed to support and decelerate the centre of mass is stored as strain energy in the tendon. Since during the propulsion phase the VL did not contribute to the forward acceleration of the body, no additional energy is necessary and a simple energy exchange within the MTU close to the optimal length of the muscle fascicles may allow for a minimisation of the activation level and duration of muscle activity during the stance phase, which improves the economy of muscle force generation. In addition, the minimal elongation of the VL fascicle may also prevent fascicles from cyclic lengthening perturbations and stretch-induced injuries^51–53^, which is essential for frequent daily life activities such as walking and running.

To determine the VL muscle fascicle length we used two-dimensional ultrasound imaging. Consequently, fascicle length would be underestimated in case were the analysed fascicles were not aligned with the image plane. Studies investigating the validity of the ultrasound-based fascicle length determination reported an acceptable validity for VL and vastus intermedius with a general systematic underestimation of the VL fascicle length, i.e. average differences of ≈6%^54^. Our systematic reliability assessment of the ultrasound-based measurement of the VL fascicle length behaviour throughout the entire step cycle revealed excellent reliability. The within-day CMCs were above 0.95 for both walking and running and RMSD values were between 7 and 8 mm. These values are comparable to those reported earlier in reliability studies on the gastrocnemius medialis muscle during walking and running^55,56^. The authors of those studies recommended assessing 6 to 9 gait trials for a reliable and accurate fascicle length determination. In the current study, we measured the VL fascicle length for 6 to 11 steps for each participant and gait, which is in line with those recommendations. We measured the VL fascicle length using an ultrasound device with a linear array probe that was longer (i.e. 10 cm) compared to most of the commonly used ultrasound systems. The 10 cm probe is very important for the accurate measurement of long fascicles as in the VL because almost the whole fascicle is visible in the image. However, due to technical limitations the maximum capturing frequency with this probe is 43 Hz, which may introduce some uncertainty in the measurement of the VL fascicle behaviour. The average duration of the active state during stance in our study was 201 ms for walking and 136 ms for running, indicating in average 9 and 6 analyzed frames, respectively. However, the test-retest analysis of the fascicle length provided an excellent reliability between single trials (i.e. overall CMC for walking 0.905 ± 0.048 and for running 0.921 ± 0.063). Therefore, in our opinion the recording frequency of 43 Hz was sufficient to provide representative VL fascicle lengths during the investigated walking and running task. We measured the EMG activity of the VL muscle in a separate cohort due to experimental constrains and this is a limitation of the current study. However, studies that investigated the VL EMG activity reported relatively consistent activation profiles among different participants relative to the step cycle^57,58^. To further account for inherent temporal gait variability between the two groups, the stance and swing times were time-normalised.

In conclusion, we were able to provide first-time evidence that (a) the fascicles of human VL operate almost isometrically and close to their optimal length during walking and running and that (b) this favourable condition for force generation was likely the result of an adjusted activation. In opposite to common belief and musculoskeletal model predictions, the findings further indicate an important contribution of tendon compliance to the fascicle length behaviour of proximal leg muscles as the VL during daily life activities.
